# Association between ALDH2 rs671 polymorphism and susceptibility to non-valvular atrial fibrillation in a Chinese population: a large-scale case–control study

**DOI:** 10.3389/fphys.2025.1669815

**Published:** 2026-02-03

**Authors:** Fangming Zhong, Qifeng Zhang, Zhuolian Ye, Xuebo He, Junping Huang, Leiming Miao

**Affiliations:** Department of Cardiology, Meizhou People’s Hospital, Meizhou, Guangdong, China

**Keywords:** ALDH2 polymorphism, non-valvular atrial fibrillation, genetic susceptibility, rs671 SNP, Chinese population

## Abstract

Non-valvular atrial fibrillation (NVAF) is the most common arrhythmia worldwide, with a steadily rising incidence and prevalence, posing a significant public health burden. Oxidative stress is recognized as a key driver of atrial remodeling and arrhythmogenesis. Mitochondrial aldehyde dehydrogenase 2 (ALDH2) plays a critical role in detoxifying reactive aldehydes, and the rs671 single-nucleotide polymorphism (G→A, Glu504Lys) markedly reduces enzymatic activity, with a high prevalence in East Asian populations. In this retrospective study, we analyzed 403 NVAF patients and 14,326 hospitalized controls from Meizhou People’s Hospital (2016–2020), aged ≥30 years (1:35.5 ratio), and constructed multiple propensity score-matched cohorts (1:15 to 1:2) to examine the association between ALDH2 rs671 and NVAF. The A allele frequency was significantly higher in NVAF patients than in the controls (32.0% vs. 24.2%, P < 0.001), causing an increased NVAF risk (OR = 1.472, 95% CI: 1.266–1.711). Multivariate logistic regression identified the GA genotype (OR = 1.681, 95% CI: 1.360–2.078, P < 0.001) and the AA genotype (OR = 1.558, 95% CI: 1.058–2.296, P = 0.025) as independent risk factors. Sensitivity analyses across various matching ratios confirmed the robustness of the association. Other independent risk factors included male sex, advanced age, hypertension, diabetes, coronary heart disease, and COPD.

## Introduction

Atrial fibrillation (AF) is the most common arrhythmia worldwide, with an annually increasing incidence and prevalence. It is projected that by 2025, at least 72 million people in Asia will be affected by AF, and approximately 3 million will suffer AF-related strokes, contributing to a considerable burden on healthcare systems (Chiang et al., 2013; Schnabel et al., 2015; Chugh et al., 2014; Dong et al., 2023; Lippi et al., 2021; Tse et al., 2013).

Despite considerable advances in clinical management, the underlying mechanisms contributing to the onset and maintenance of non-valvular atrial fibrillation (NVAF) remain incompletely understood. The known risk factors include advanced age, male sex, hypertension, diabetes mellitus, left ventricular dysfunction, obesity, chronic obstructive pulmonary disease (COPD), alcohol consumption, and genetic predisposition (Lubitz et al., 2010; Roselli et al., 2018).

Recent studies have increasingly implicated oxidative stress in the pathogenesis of AF (Xu et al., 2017; Yang et al., 2018; Seo et al., 2019; Suo et al., 2019; Sung et al., 2016). Reactive oxygen species (ROS), through their effects on ion channel remodeling, intercellular coupling, and mitochondrial function, can induce electrical and structural remodeling of atrial tissue, thereby facilitating arrhythmogenesis. Among the regulatory pathways of ROS homeostasis, mitochondrial aldehyde dehydrogenase 2 (ALDH2) plays a central role by detoxifying reactive aldehydes such as 4-hydroxynonenal, which is a major byproduct of lipid peroxidation. Impaired ALDH2 activity may exacerbate oxidative damage and trigger atrial remodeling, which contributes to AF development.

ALDH2 is also a critical enzyme in alcohol metabolism, and its most clinically significant genetic variant is the rs671 single-nucleotide polymorphism (SNP), in which guanine (G) is replaced by adenine (A), resulting in a Glu504Lys amino acid substitution. This mutation defines three genotypes, namely, GG (normal enzyme activity), GA (moderately reduced activity), and AA (negligible activity) (Mizoi et al., 1994). The A allele is associated with impaired clearance of acetaldehyde and heightened oxidative stress, both of which have been linked to cardiovascular pathologies.

Although carriers of the rs671 A allele have been shown to be at elevated risk for multiple systemic diseases, including cardiovascular and cerebrovascular disorders (Mizoi et al., 1994; Chen et al., 2019; Yang et al., 2020; Purohit et al., 2013; Liu et al., 2019; Emelyanova et al., 2016; Lin et al., 2003; Mihm et al., 2001; Gao et al., 2022; Zhu et al., 2022; Sung et al., 2016; Nannelli et al., 2018; Zhong et al., 2019), its association with atrial fibrillation—particularly NVAF—has not been systematically investigated in the Chinese population. Given the dual role of ALDH2 in acetaldehyde metabolism and ROS detoxification, this study aims to evaluate the potential relationship between ALDH2 rs671 polymorphism and susceptibility to NVAF. We further aim to identify whether ALDH2 variants represent a genetic risk factor and provide new insights into the prevention and individualized management of NVAF in East Asian populations.

## Materials and methods

### Study population

**FIGURE 1:**
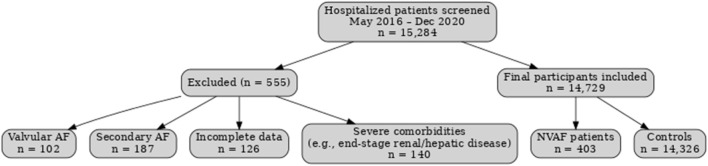
Participant flow diagram.

This retrospective case–control study included patients with NVAF treated at Meizhou People’s Hospital between 1 May 2016 and 31 December 2020, along with hospitalized patients aged ≥30 years without NVAF during the same period, who served as the controls. A total of 14,729 individuals were enrolled (403 NVAF cases and 14,326 controls), with a full-sample ratio of approximately 1:35.5. A detailed flow diagram of participant enrollment, exclusion, and final numbers analyzed is shown in Figure 1. The exclusion criteria were defined as follows: patients aged less than 30 years; those with valvular heart disease, congenital heart disease, or severe structural heart disease; those with secondary AF caused by hyperthyroidism, acute myocardial infarction, or pulmonary embolism; those with incomplete baseline clinical data; and those with poor-quality or unavailable DNA samples.

To avoid large increases in matching ratios and to evaluate robustness across a smoother gradient of case–control balance, we additionally constructed propensity score-matched (PSM) cohorts at the following ratios: 1:15, 1:10, 1:8, 1:6, 1:4, 1:3, and 1:2 (case : control). Matching was performed using nearest neighbor without replacement and a caliper of 0.2 standard deviations on the logit of the propensity score, with sex being exact-matched. Covariate balance was assessed using standardized mean differences (SMD < 0.10 as the threshold), and multivariable logistic regression was applied to each matched dataset to estimate the adjusted odds ratios (ORs) and 95% confidence intervals (CIs). This gradient—1:35.5 → 1:15 → 1:10 → 1:8 → 1:6 → 1:4 → 1:3 → 1:2—ensures progressive improvement in comparability while maintaining adequate power at higher ratios. The research scheme was approved by the Ethics Committee of Meizhou People’s Hospital and is in accordance with the guidelines of the Helsinki Declaration (Approval No.: 2020-C-105). Data such as baseline demography, medical history, and AF history were mainly collected from the patient’s past medical records. Data collection and analysis were conducted anonymously, and there was no disclosure of patient privacy information; therefore, there was no need for informed consent.

### Definitions

NVAF is defined as AF occurring in the absence of moderate-to-severe mitral stenosis or mechanical heart valves. NVAF also meets the following general diagnostic criteria for AF: an episode lasting more than 30 s that is not attributable to reversible causes. On electrocardiography, NVAF is characterized by the absence of P waves, which are replaced by fibrillatory (f) waves with completely irregular amplitude, morphology, and intervals (350/min–600/min), along with an absolutely irregular ventricular response. Patients whose AF was due to reversible causes—such as acute myocardial infarction, acute myocarditis, acute pulmonary embolism, untreated hyperthyroidism, AF induced by electrophysiological examination, angiography, pacemaker implantation, or recent cardiothoracic surgery—were excluded from the study (Hindricks et al., 2021).

Control group criteria: Controls were defined as patients aged ≥30 years who were hospitalized during the study period and met all of the following criteria, identified by medical record review: (1) no documented history or electrocardiographic evidence of AF (any type); (2) absence of moderate or severe valvular heart disease; (3) absence of notable structural heart disease (e.g., hypertrophic cardiomyopathy and dilated cardiomyopathy); (4) absence of other major sustained arrhythmias (e.g., sustained ventricular tachycardia and atrial flutter); (5) absence of a primary admission diagnosis related to an acute cardiovascular event (e.g., acute myocardial infarction, acute heart failure exacerbation, or acute pulmonary embolism); (6) absence of documented severe heart failure (NYHA class III/IV), end-stage liver disease, or end-stage renal disease that requires dialysis.

Control group exclusion criteria: (1) Any documented history or electrocardiographic evidence of AF (any type); (2) moderate or severe valvular heart disease; (3) notable structural heart disease (e.g., hypertrophic cardiomyopathy and dilated cardiomyopathy); (4) other major sustained arrhythmias (e.g., sustained ventricular tachycardia and atrial flutter); (5) primary admission diagnosis related to an acute cardiovascular event (e.g., acute myocardial infarction, acute heart failure exacerbation, and acute pulmonary embolism); (6) documented severe heart failure (NYHA class III/IV), end-stage liver disease, or end-stage renal disease that requires dialysis; (7) age lesser than 30 years; (8) incomplete medical records preventing accurate control group classification.

Information on the age, gender, hypertension, diabetes, coronary heart disease, stroke, COPD, heart failure, hyperthyroidism, and ALDH2 genotypes was collected from medical records. Hypertension was defined based on the following criteria: systolic blood pressure ≥ 140 mmHg and/or diastolic blood pressure ≥ 90 mmHg, use of antihypertensive drugs, or self-reported diagnosis of hypertension by a doctor. Diabetes was defined based on the following criteria: fasting blood glucose ≥ 7.0 mmol/L, postprandial blood glucose ≥ 11.1 mmol/L or glycosylated hemoglobin ≥ 7%, use of antidiabetic drugs, or diagnosis by a doctor. Coronary heart disease was defined based on the following criteria: stable angina pectoris, unstable angina pectoris, myocardial infarction (MI), percutaneous coronary intervention, or coronary artery bypass grafting history. Stroke was defined as any acute attack, transient ischemic attack (TIA), or permanent neurological dysfunction diagnosed or reported by a doctor. COPD was defined as irreversible obstructive ventilatory dysfunction diagnosed by a doctor or indicated by pulmonary function. Heart failure was defined as that measured by color Doppler ultrasound (EF < 40%), use of anti-heart failure drugs, or a self-reported heart failure diagnosed by a doctor. Hyperthyroidism was defined based on the following criteria: use of antihyperthyroidism drugs, I^131^ treatment, thyroidectomy, or diagnosis by a doctor.

### Genotyping of ALDH2 polymorphism

For each subject, 3 mL of EDTA-anticoagulated peripheral blood was drawn within the first 24 h after the initial hospital admission and before the initiation of any anti-arrhythmic, anticoagulant, or interventional therapy (e.g., electrical cardioversion or catheter ablation). Samples were processed within 2 h, aliquoted, and stored at −80 °C in the institutional biobank until DNA extraction. Sampling time stamps and treatment records were retrieved from the electronic medical record to confirm the pretreatment status.

Genomic DNA was extracted from peripheral blood samples using the TIANGEN Blood Genomic DNA Extraction Kit (TIANGEN Biotech, Beijing, China) according to the manufacturer’s protocol. DNA concentration and purity were measured with a NanoDrop 2000 spectrophotometer (Thermo Scientific, Waltham, USA), and only samples with an A260/A280 ratio between 1.8 and 2.0 were included for further analysis.

Specific primers for ALDH2 gene polymorphism were designed using Primer Premier 5.0 software (Premier Biosoft International, Palo Alto, USA). PCR amplification was performed in a 25-μL reaction mixture under optimized conditions. PCR products were analyzed by 2% agarose gel electrophoresis and stained with GoldView™ nucleic acid stain (Solarbio, Beijing, China) to verify the expected fragment size.

The amplified PCR products were then subjected to bidirectional Sanger sequencing, which was commercially performed by GeneChem Co., Ltd. (Hangzhou, China) to determine the genotypes. Genotyping analyses were performed in two independent batches, with case and control samples randomly assigned to each batch to minimize batch effects.

Quality control procedures included blinded duplicate genotyping for 10% of the samples randomly selected from the study population, and the concordance rate was above 99%. Each PCR and sequencing batch contained both negative (no template control) and positive controls to monitor for contamination and provide technical validation. PCR primers and conditions were validated through preliminary experiments to ensure specificity and reproducibility. Additionally, Hardy–Weinberg equilibrium (HWE) was evaluated in the control group for each SNP.

### Statistical analysis

The propensity score was estimated using a logistic regression model including the following baseline variables: age, sex, hypertension, diabetes mellitus, and coronary heart disease. These covariates were selected because they are established risk factors for AF and were consistently available in our dataset.

We analyzed the association between ALDH2 rs671 polymorphism and NVAF under an additive genetic model, which assumes a linear trend in risk with the increasing number of A alleles (coded as 0 = GG, 1 = GA, and 2 = AA). Logistic regression models were constructed accordingly. All data were analyzed using SPSS version 25.0. Measurement data are expressed as the mean ± standard deviation (x̄ ± s), and categorical data are expressed as the frequency and percentage. Continuous variables between two groups were compared using the independent-sample t-test, and categorical variables were compared using the χ^2^ test. The representativeness of the case and control groups was assessed by HWE, with P > 0.05 indicating genetic equilibrium. The study population was derived from the same Mendelian population. Binary logistic regression was used to evaluate the risk factors, and ORs with 95% CIs were calculated. A two-sided P < 0.05 was considered statistically significant.

To further evaluate the robustness of the association between ALDH2 rs671 polymorphism and NVAF, we performed a series of sensitivity analyses. First, in the entire cohort (n = 14,729), both univariate and multivariate logistic regression models were applied. Multivariate models were sequentially adjusted for potential confounders:

Model 1: adjusted for age and sex.
Model 2: additionally adjusted for hypertension, diabetes, and coronary heart disease.
Model 3: further adjusted for COPD and stroke history.

Second, we conducted propensity score matching (PSM) to minimize baseline differences between NVAF patients and controls. Matching variables included age, sex, hypertension, diabetes, and coronary heart disease. Four PSM datasets were generated using matching ratios of 1:15, 1:10, 1:5, and 1:2. Within each matched dataset, three logistic regression models were constructed:

A crude model without covariate adjustment.
A model adjusted for age and sex.
A fully adjusted model including the unmatched variables or those with residual confounding.

Results from the entire cohort are presented in Figure 2, whereas results from the PSM cohorts are summarized in Figures 3–9. This step-wise approach allowed us to assess consistency across unadjusted and adjusted models and between unmatched and matched samples.

**FIGURE 2:**
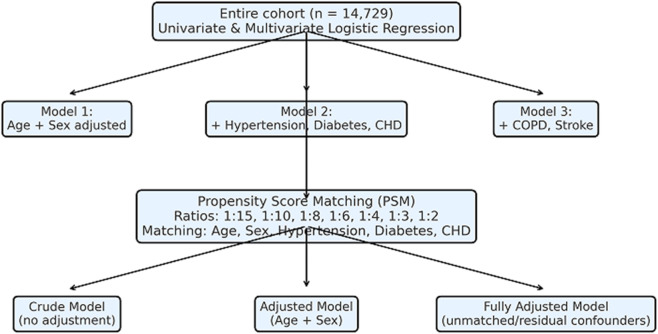
Logistic regression analysis for risk factors in AF patients.

**FIGURE 3:**
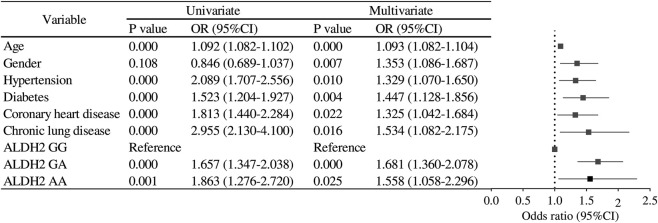
Logistic regression analysis for risk factors in AF patients.

**FIGURE 4:**
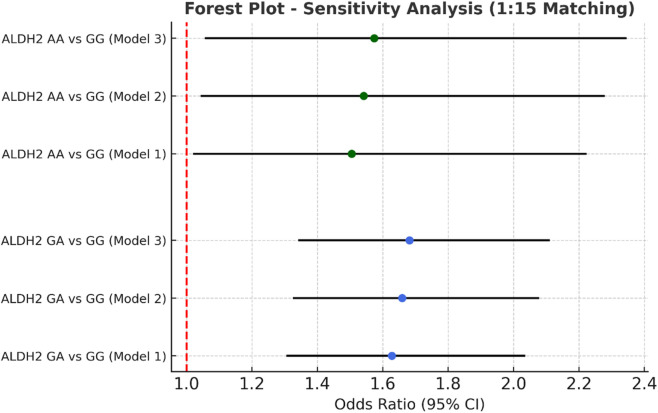
Sensitivity analysis multivariate logistic regression models with varying covariate sets (1:15).

**FIGURE 5:**
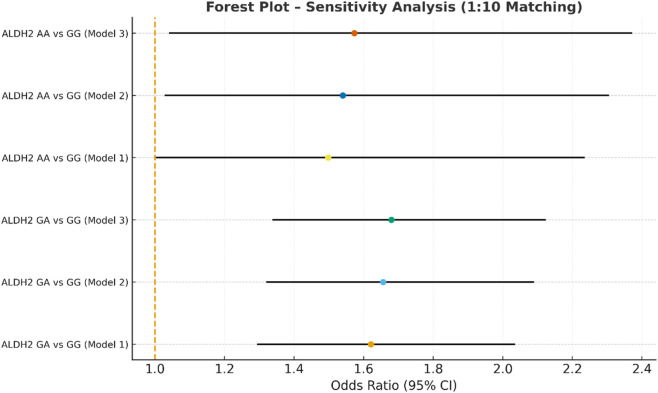
Sensitivity analysis multivariate logistic regression models with varying covariate sets (1:10).

**FIGURE 6:**
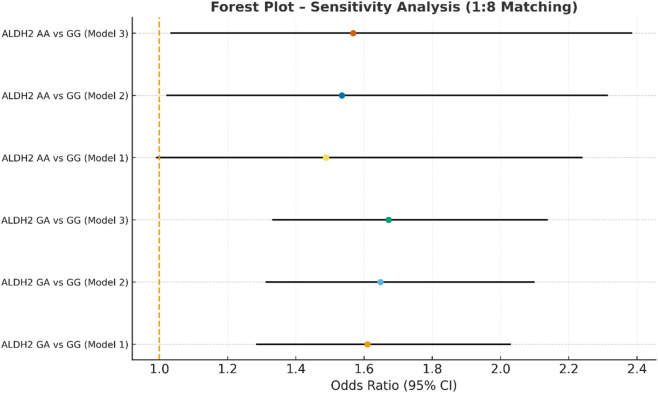
Sensitivity analysis multivariate logistic regression models with varying covariate sets (1:8).

**FIGURE 7:**
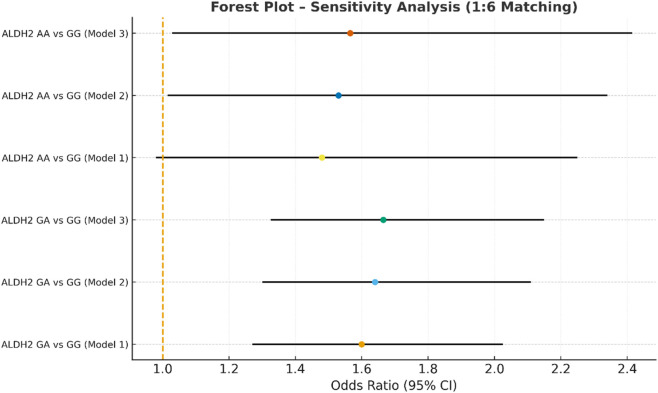
Sensitivity analysis multivariate logistic regression models with varying covariate sets (1:6).

**FIGURE 8:**
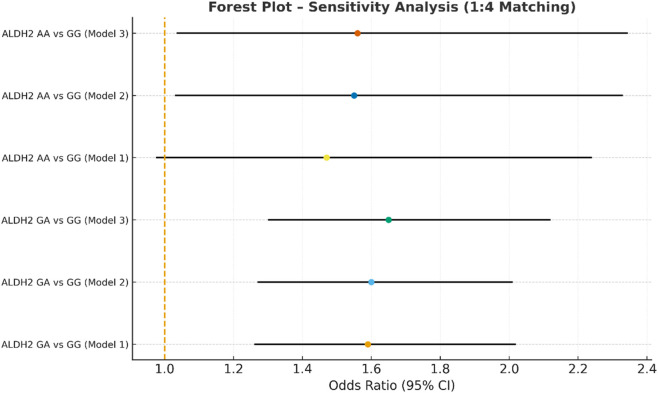
Sensitivity analysis multivariate logistic regression models with varying covariate sets (1:4).

**FIGURE 9:**
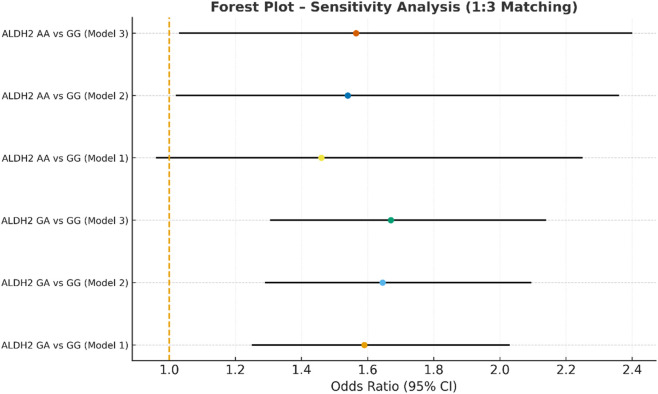
Sensitivity analysis multivariate logistic regression models with varying covariate sets (1:3).

### Design justification and bias control

To justify the initial unbalanced 1:35.5 case–control ratio, we included all the consecutively identified NVAF cases and all eligible hospitalized controls during the study period. Although this deviates from conventional matching designs, such an inclusive strategy offers two methodological advantages for genetic association studies: (1) maximized statistical power to detect modest effect sizes (e.g., OR < 1.5), particularly for rare genotypes such as ALDH2 AA, and (2) precise estimation of allele frequencies in the background population.

To mitigate potential confounding from this sampling imbalance and evaluate the robustness of the findings under varying degrees of case–control balance, we conducted additional propensity score-matched analyses across a series of progressively balanced ratios: 1:15, 1:10, 1:8, 1:6, 1:4, 1:3, and 1:2 (case : control). With nearest-neighbor matching without replacement and a 0.2 SD caliper on the logit of the propensity score, sex was exact-matched. Covariate balance was assessed using standardized mean differences (SMD < 0.10). For each ratio, multivariable logistic regression adjusted for any unmatched or residual-imbalance covariates was applied to obtain adjusted ORs and 95% CIs.

This multi-ratio gradient—1:35.5 → 1:15 → 1:10 → 1:8 → 1:6 → 1:4 → 1:3 → 1:2—allowed us to assess the stability of effect estimates across progressively stricter comparability, minimize selection bias, and maintain adequate statistical power in higher-ratio designs. These methodological choices collectively enhance the internal validity, robustness, and generalizability of our findings.

## Results

### Demographic characteristics and clinical data of the NVAF group

We analyzed 403 patients with NVAF and 14,326 control subjects. The demographic and clinical characteristics of the NVAF group are presented in Table 1. Among the different ALDH2 genotypes in the NVAF group, there were no significant differences in age, sex, body mass index (BMI), heart failure, hypertension, diabetes, stroke, coronary heart disease, COPD, cardiac color Doppler ultrasound findings, or CHA2DS2-VASc scores.

**TABLE 1:** Demographical characteristics and clinical data of the NVAF group.

Variable	GG (n = 178)	GA (n = 192)	AA (n = 33)	P-value
Demographics				
Age (y, mean ± SD)	74.6 ± 11.3	73.9 ± 10.1	77.9 ± 8.3	0.162
Gender (% male)	103 (57.9%)	125 (65.1%)	21 (61.8%)	0.350
BMI (kg/m^2^)	23.3 ± 3.4	23.3 ± 3.1	23.4 ± 3.4	0.994
Comorbidities (%)				
Heart failure	32 (18.0%)	23 (12.0%)	4 (12.1%)	0.241
Hypertension	107 (60.1%)	115 (59.9%)	17 (59.3%)	0.636
Diabetes mellitus	37 (20.8%)	49 (25.5%)	9 (23.6%)	0.491
Stroke	56 (31.5%)	74 (38.5%)	15 (45.5%)	0.182
Vascular disease	46 (25.8%)	44 (22.9%)	10 (30.3%)	0.605
Chronic lung disease	13 (7.3%)	25 (13%)	5 (15.2%)	0.140
Echocardiographic characteristics				
LA (mm)	40.2 ± 7.0	39.5 ± 8.3	38.4 ± 6.2	0.374
IVS (mm)	10.6 ± 1.4	10.7 ± 1.6	10.8 ± 1.8	0.662
EF (%)	53.9 ± 12.5	54.3 ± 12.3	53.3 ± 12.2	0.679
CHA2DS2-VASc score	3.35 ± 1.54	3.30 ± 1.53	3.67 ± 1.59	0.456

Abbreviations: BMI, body mass index; LVEF, left ventricular ejection fraction.

### Genotype and allele distributions in NVAF patients and controls

The distributions of the ALDH2 genotypes and allele frequencies in the NVAF group and control group are shown in Table 2. The distributions of the ALDH2 (rs671) genotypes in the NVAF group (χ^2^ = 3.60, P = 0.06) and the control group (χ^2^ = 1.32, P = 0.25) were in HWE equilibrium, indicating that all the selected subjects were representative of the population (P > 0.05). The proportions of ALDH2 GG, GA, and AA genotypes in the NVAF group were 44.2%, 47.6%, and 8.2%, respectively. The G and A allele frequencies were 68.0% and 32.0%, respectively. In the control group, the frequencies of the ALDH2 GG, GA, and AA genotypes were 57.2%, 37.1%, and 5.7%, respectively, and the frequencies of the G and A alleles were 75.8% and 24.2%, respectively. The frequency of allele A in patients with NVAF was significantly higher than that in the control group (32.0% vs. 24.2%, P < 0.001). Relative risk analysis showed that individuals with allele A had a higher risk of developing NVAF (OR = 1.472, 95% CI = 1.266–1.711, P < 0.001). We further analyzed a variety of genetic models. ① The recessive model (ALDH2 GG vs. ALDH2 GA + AA) used the normal homozygous ALDH2 GG genotype as a reference, and relative risk analysis showed that individuals with ALDH2 GA + AA genotypes had an increased risk of NVAF (OR = 1.692, 95% CI = 1.386–2.065, P < 0.001). ② The homozygous model (ALDH2 GG vs. ALDH2 AA) used the homozygous ALDH2 GG genotype as a reference, and relative risk analysis showed that individuals with the ALDH2 AA genotype had an increased risk of developing NVAF (OR = 1.863, 95% CI = 1.276–2.720, P = 0.001). ③ The additive model (ALDH2 GG vs. ALDH2 GA) used the homozygous ALDH2 GG genotype as a reference, and relative risk analysis showed that individuals with the ALDH2 GA genotype had an increased risk of NVAF (OR = 1.665, 95% CI = 1.354–2.048, P < 0.001). However, there was no significant difference in the genotype frequency between NVAF patients and controls in the dominant model (ALDH2 GG + GA genotypes vs. ALDH2 AA genotype) or the heterozygous model (ALDH2 GA genotype vs. ALDH2 AA genotype).

**TABLE 2:** ALDH2 polymorphism in AF patients and controls.

SNP	Model	Genotype	AF	Control	P-value	OR	95% CI
ALDH2	Allele	G	548 (68.0%)	21,709 (75.8%)		1.000	Reference
		A	258 (32.0%)	6,943 (24.2%)	0.000	1.472	1.266–1.711
	Dominant	GG + GA	370 (91.8%)	13,510 (94.3%)		1.000	Reference
		AA	33 (8.2%)	816 (5.7%)	0.034	1.477	1.027–2.123
	Recessive	GG	178 (44.2%)	8,199 (57.2%)		1.000	Reference
		GA + AA	225 (55.8%)	6,127 (42.8%)	0.000	1.692	1.386–2.065
	Heterozygote	GA	192 (85.3%)	5,311 (86.7%)		1.000	Reference
		AA	33 (14.7%)	816 (13.3%)	0.559	1.119	0.768–1.630
	Homozygote	GG	178 (84.4%)	8,199 (90.9%)		1.000	Reference
		AA	33 (15.6%)	816 (9.1%)	0.001	1.863	1.276–2.720
	Additive	GG	178 (48.1%)	8,199 (60.7%)		1.000	Reference
		GA	192 (51.9%)	5,311 (39.3%)	0.000	1.665	1.354–2.048
	HW test		χ^2^ = 3.60 P = 0.06	χ^2^ = 1.32 P = 0.25			

SNP, single-nucleotide polymorphism; OR, odds ratio; CI, confidence interval; HWE, Hardy–Weinberg equilibrium.

### Risk factors identified for NVAF patients by univariate and multivariate regression

Univariate and multivariate logical regression analyses were performed to identify independent and significant variables affecting NVAF (Figure 3). Multivariate logistic regression analysis, after adjusting for the identified risk factors (gender, age, hypertension, diabetes, coronary heart disease, and COPD), showed that the ALDH2 GA genotype carriers and ALDH2 AA genotype carriers had a higher risk of NVAF than those carrying the ALDH2 GG genotype, suggesting that the ALDH2 GA genotype and ALDH2 AA genotype were independent risk factors for NVAF (ALDH2 GA genotype OR = 1.681, 95% CI: 1.360–2.078, P < 0.001; ALDH2 AA genotype OR = 1.558, 95% CI: 1.058–2.296, P = 0.025). For other covariates, male sex, advanced age, hypertension, diabetes, coronary heart disease, and COPD were independent risk factors for NVAF (all P < 0.05).

### Sensitivity analyses of propensity score-matched cohorts

We found that the A allele was significantly associated with increased NVAF susceptibility (OR = 1.472, 95% CI: 1.266–1.711, P < 0.001), and both the GA and AA genotypes were independent risk factors in multivariate-adjusted models (GA: OR = 1.681, 95% CI: 1.360–2.078, P < 0.001; AA: OR = 1.558, 95% CI: 1.058–2.296, P = 0.025).

**FIGURE 10:**
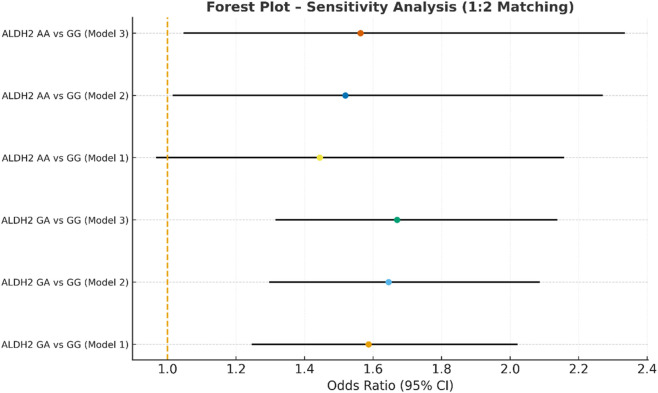
Sensitivity analysis multivariate logistic regression models with varying covariate sets (1:2).

In the sensitivity analysis, we further incorporated propensity score-matched results at different case–control ratios to verify the robustness of the association between the ALDH2 rs671 genotype and NVAF. In addition to the full-sample analysis (1:35.5), we sequentially constructed matched cohorts at ratios of 1:15, 1:10, 1:8, 1:6, 1:4, 1:3, and 1:2 with matching covariates including age, sex, hypertension, diabetes, coronary heart disease, and COPD, ensuring that the standardized mean differences (SMD) for all covariates were controlled within 0.10 across all ratios (Figures 4–10). Quantitatively, regardless of the case–control ratio applied, the ORs for the GA and AA genotypes relative to the GG genotype remained similar in magnitude and consistent in direction, indicating the stability of the observed association. For example, for the GA genotype, the model 2-adjusted ORs at ratios of 1:15, 1:10, 1:8, 1:6, 1:4, 1:3, and 1:2 were 1.659 (95% CI: 1.325–2.078), 1.656 (1.320–2.090), 1.648 (1.311–2.100), 1.640 (1.300–2.110), 1.600 (1.270–2.010), 1.645 (1.290–2.095), and 1.645 (1.296–2.087), respectively. The variations were minimal, and all the results reached statistical significance (P < 0.001), indicating that the GA genotype was an independent risk factor under different matching conditions. Similarly, for the AA genotype, the model 2-adjusted ORs across the multiple matching ratios ranged from 1.519 to 1.542; although the CIs were slightly wider and some ratios approached the significance threshold, the overall trend was consistent with that of the GA genotype, supporting the susceptibility effect of the A allele. Qualitatively, as the case–control ratio became more balanced (e.g., 1:2 matching), the precision of the effect estimates decreased and the 95% CIs widened, which was attributable to reduced statistical power due to smaller sample sizes; however, the direction of the association remained unchanged. These findings indicate that the relationship between ALDH2 polymorphism and NVAF risk is highly robust and reproducible.

## Discussion

AF is the most common arrhythmia and poses a significant global public health challenge due to its increasing prevalence and the associated social and economic burden (Schnabel et al., 2015; Chugh et al., 2014; Dong et al., 2023; Lippi et al., 2021). Although the pathogenesis of NVAF remains incompletely understood, accumulating evidence suggests that both genetic and environmental factors are involved.

### ALDH2 and oxidative stress in atrial remodeling

Mitochondrial oxidative stress is considered a major contributor to the structural and electrophysiological remodeling that underlies AF. ALDH2, a key enzyme in mitochondrial metabolism, plays an essential role in detoxifying reactive aldehydes such as 4-hydroxynonenal (4-HNE) (Santin et al., 2020), a lipid peroxidation byproduct known to accumulate in atrial tissue during AF. Dysfunction of ALDH2 impairs the clearance of these toxic aldehydes and exacerbates oxidative stress, which has been shown to contribute to ion channel dysregulation, mitochondrial injury, and myocyte apoptosis (Emelyanova et al., 2016; Ebert et al., 2014; Li et al., 2018; Zhao et al., 2019). Previous studies have demonstrated that atrial tissue in AF patients shows elevated ROS levels and increased oxidative damage (Emelyanova et al., 2016; Lin et al., 2003; Mihm et al., 2001). *In vitro* models further support that ALDH2 deficiency promotes oxidative damage and cardiomyocyte dysfunction (Ebert et al., 2014). Therefore, impaired ALDH2 function may create a pro-arrhythmic substrate via oxidative injury.

### rs671 variant and population-specific metabolic risk

The rs671 polymorphism in ALDH2, involving a G-to-A substitution leading to a Glu504Lys amino acid change, is the most functionally significant variant in East Asian populations. This mutation gives rise to three genotypes: GG (normal activity), GA (activity reduced by ∼80–90%), and AA (almost complete loss of activity) (Mizoi et al., 1994). As a result, carriers of the A allele have impaired acetaldehyde metabolism and are more susceptible to aldehyde-induced toxicity and oxidative stress. In the Chinese population, the frequencies of the GA and AA genotypes are approximately 34.27% and 4.50%, respectively—significantly higher than those in European and African populations (Cao et al., 2020). This high prevalence suggests that over one-third of the Chinese population carries a reduced-function variant of ALDH2.

Our findings are consistent with these distribution patterns. In our cohort, the allele and genotype frequencies of ALDH2 rs671 closely mirrored those reported in Japanese and Korean populations, where the A allele frequencies ranged from 0.16 to 0.28 (Kim et al., 2021). This cross-ethnic consistency enhances the external validity of our study within East Asian populations.

### Comparison with previous studies and our findings

In addition, our findings should be interpreted in the context of prior research. A recent study examined the association of ALDH2 rs671 polymorphism with the occurrence and progression of AF and reported that individuals with the wild-type GG genotype exhibited a greater AF burden compared with GA or AA carriers, which the authors attributed to increased alcohol consumption (Ge et al., 2022; Yan et al., 2024). Although our study identified the A allele as a genetic risk factor for NVAF susceptibility, these differences may be explained by population characteristics, study design, and the absence of detailed alcohol exposure data in our cohort. Taken together, the results highlight the importance of considering gene–environment interactions, particularly alcohol consumption, when evaluating the role of ALDH2 variants in AF risk.

### Clinical implications and future research

Given the dual role of ALDH2 in aldehyde detoxification and oxidative stress regulation, our findings provide genetic and mechanistic evidence linking rs671 to NVAF risk in East Asians. From a clinical standpoint, this highlights the potential utility of incorporating ALDH2 genotyping into personalized AF risk prediction models. Individuals with the GA or AA genotype may benefit from targeted screening, lifestyle interventions, and antioxidant therapies aimed at mitigating atrial oxidative stress.

Nonetheless, this study has limitations. First, its retrospective single-center design introduces potential selection bias. Second, detailed data on alcohol consumption were unavailable, making it difficult to distinguish between direct genetic effects and alcohol-mediated risk. Future studies should stratify patients by drinking behavior to address this. Third, our findings may not be generalizable for populations beyond southern China or across ethnic subgroups within China. Therefore, multi-center, prospective cohort studies are required to confirm these associations and evaluate gene–environment interactions in diverse populations.

## Conclusion

This large-scale Chinese population study demonstrates a significant association between the ALDH2 rs671 A allele and NVAF susceptibility, with increased risk among the GA and AA genotype carriers. ALDH2 genotyping may serve as a genetic reference for identifying high-risk individuals, particularly in East Asian populations.

## Highlights

We investigated whether the ALDH2 rs671 variant is associated with susceptibility to NVAF in a Chinese hospital-based cohort. Using PCR and bidirectional Sanger sequencing, we genotyped 403 NVAF cases and 14,326 controls aged ≥30 years and analyzed the association with multivariable logistic regression and propensity score-matched cohorts. The rs671 A-allele showed a higher frequency in NVAF cases than in controls and was associated with increased NVAF risk, and both the GA and AA genotypes remained significant after adjustment. Results were consistent across matching ratios. This evidence supports a link between impaired aldehyde detoxification, oxidative stress, and atrial arrhythmogenesis and suggests that ALDH2 genotyping may help refine risk stratification in East Asian populations. The main limitations include the retrospective single-center design and the lack of detailed alcohol-exposure data.

## Data Availability

The original contributions presented in the study are included in the article/supplementary material; further inquiries can be directed to the corresponding author.
